# Variants of Tn*6924*, a Novel Tn*7* Family Transposon Carrying the *bla*_NDM_ Metallo-β-Lactamase and 14 Copies of the *aphA6* Amikacin Resistance Genes Found in Acinetobacter baumannii

**DOI:** 10.1128/spectrum.01745-21

**Published:** 2022-01-12

**Authors:** Riti Mann, Rayane Rafei, Cindy Gunawan, Christopher J. Harmer, Mohammad Hamidian

**Affiliations:** a The iThree Institute, University of Technology Sydney, Ultimo, New South Wales, Australia; b Laboratoire Microbiologie Santé et Environnement (LMSE), Doctoral School of Science & Technology, Faculty of Public Health, Lebanese University, Tripoli, Lebanon; c School of Chemical Engineering, University of New South Wales, Sydney, New South Wales, Australia; d School of Life and Environmental Sciences, The University of Sydney, Sydney, New South Wales, Australia; University of Manitoba

**Keywords:** *Acinetobacter baumannii*, aminoglycoside resistance, carbapenem resistance, *aphA6*, *bla*_NDM_, antibiotic resistance, Tn*6924*

## Abstract

Carbapenem resistance in Acinetobacter baumannii is primarily due to the global spread of two main clones that carry *oxa23*, *oxa24*, and *oxa58.* However, new carbapenem-resistant clones are emerging that are also resistant to a wide range of antibiotics. Strains belonging to ST85_IP_ (Institut Pasteur) carry the *bla*_NDM_ metallo-β-lactamase carbapenem resistance gene. Here, we completed the genome sequence of an ST85_IP_ strain, Cl300, recovered in 2015 in Lebanon, using a combination of Illumina MiSeq and Oxford Nanopore sequencing and a hybrid assembly approach. Cl300 is highly resistant to meropenem and amikacin, and consistent with this, a copy of the *bla*_NDM_ carbapenem and 14 copies of the *aphA6* amikacin resistance genes were found in the genome. Cl300 also contains the *sul2* sulfonamide and the *msr*(E) macrolide resistance genes. All *aphA6* copies and *bla*_NDM_ are in a novel 76-kb Tn*7* family transposon designated Tn*6924*. Like Tn*7*, Tn*6924* is bounded by 29-bp inverted repeats with additional TnsB binding sites at each end. Several variants of Tn*6924* were found in a set of diverse strains, including ST85_IP_ strains as well as members of global clones 1 and 2. *sul2* and *msr*(E) are in a 13.0-kb pseudocompound transposon (PCT) bounded by IS*1008*. ST85s represent a diverse group of strains, particularly in their antibiotic resistance gene content and the K and OC surface polysaccharide loci. Acquisition of Tn*6924* by members of global clones indicates the significance of this transposon in spreading two clinically significant resistance genes, *bla*_NDM_ and *aphA6*.

**IMPORTANCE** To date, efforts to study the resistance mechanisms of carbapenem-resistant Acinetobacter baumannii have been largely focused on the two major globally distributed clones (GC1 and GC2). ST85 is an emerging sequence type, and unlike other clones, it is associated with the carriage of the *bla*_NDM_ gene. Here, we completed the genome sequence of an ST85 strain and showed that *bla*_NDM_ and 14 copies of the *aphA6* amikacin resistance genes are in Tn*6924*, a novel Tn*7* family transposon. Analysis of all publicly available ST85s predicted that all strains in the main lineage carry a variant of Tn*6924*. Variants of Tn*6924* were also found in other clones, including GC1 and GC2. Tn*6924* is an important mobile element given that it carries two clinically important resistance genes (*bla*_NDM_ and *aphA6*) and has spread to other clones. Therefore, outbreaks caused by ST85s should be studied and tracked.

## INTRODUCTION

Acinetobacter baumannii is a Gram-negative opportunistic pathogen that causes a range of nosocomial infections. Infections caused by this microorganism have become a challenge to treat due to high levels of antibiotic resistance (1). In particular, the global spread of carbapenem-resistant A. baumannii (CRAb) strains that are also extensively drug resistant has become a major concern (2, 3).

In A. baumannii, carbapenem resistance is predominantly caused by genes encoding the class D OXA-23, OXA-24 (OXA-40), and OXA-58 β-lactamases (oxacillinases), while class B enzymes (metallo-β-lactamases) are uncommon. The majority of CRAb strains belong to two major global clones, global clone 1 (GC1) and 2 (GC2), also known as international clones (ICs) (2, 4), with GC2s being the most prevalent clone in most geographical areas (2). In the last few years, several studies have reported the emergence of CRAb strains that do not belong to the major global clones, such as those belonging to ST25_IP_ (5–10) and, more recently, CRAb strains belonging to ST85_IP_ (3, 11–19). Although low in number compared to the widespread GC1 and GC2 isolates, ST85 strains have been reported in Algeria, France, Tunisia, Spain, Turkey, Lebanon, and Libya (3, 11–19). Notably, in most cases, carbapenem resistance in ST85_IP_ strains is associated with the presence of the *bla*_NDM_ metallo-β-lactamase gene, which encodes a class B carbapenemase enzyme (13, 15, 16, 18, 20), as well as, in some cases, genes conferring high levels of aminoglycoside resistance, in particular, to kanamycin and tobramycin (11, 19–21). However, despite their significance, the genetic context of antibiotic resistance genes, especially the *bla*_NDM_ metallo-β-lactamase gene, has not been studied in detail.

Here, we completed the genome sequence and examined the genomic context of antibiotic resistance genes in an ST85_IP_ CRAb strain recovered in Lebanon. We show that the *bla*_NDM_ and the *aphA6* (amikacin, kanamycin, and neomycin resistance) genes are in a novel Tn*7* family transposon designated Tn*6924*. Furthermore, we studied the evolution of Tn*6924* using a comparative analysis of several related transposons in publicly available complete ST85_IP_ genomes.

## RESULTS

### Antibiotic resistance profile.

Cl300 was found to be resistant to carbapenems (imipenem and meropenem), ampicillin, third-generation cephalosporins (cefotaxime and ceftazidime), streptomycin, amikacin, netilmicin, spectinomycin, neomycin, kanamycin, sulfamethoxazole, nalidixic acid, ciprofloxacin, trimethoprim, florfenicol, and chloramphenicol (Table S1 in the supplemental material). Cl300 is susceptible to colistin, with an MIC of <0.25 mg/L, and showed reduced susceptibility to ampicillin-sulbactam (Table S1).

### Complete genome sequence of Cl300 and antibiotic resistance genes.

The genome of Cl300 was completed using a combination of Illumina MiSeq and Oxford Nanopore (MinION) data using a hybrid assembly approach. The genome assembly was repeated three times and always resulted in an identical genome assembly, which consists of a 4,007,379-bp chromosome and a 9,205-bp plasmid named pCl300.

The Cl300 genome was found to contain 14 copies of the *aphA6* (amikacin resistance) gene (locus ids K9C16_00500, K9C16_00510, K9C16_00520, K9C16_00530, K9C16_00540, K9C16_00550, K9C16_00560, K9C16_00570, K9C16_00655, K9C16_00665, K9C16_00675, K9C16_00685, K9C16_00695, and K9C16_00705 in GenBank accession number CP082952) and a single copy of *bla*_NDM_ (carbapenem resistance; locus id K9C16_00580), *sul2* (sulfonamide resistance; locus id K9C16_12420), *msr*(E) (macrolide-triamilide resistance; locus id K9C16_12495) and *ble*_MBL_ (bleomycin resistance; locus id K9C16_00585), accounting for its resistance phenotype observed. The *bla*_NDM_ gene refers to the *bla*_NDM-1_ throughout the manuscript.

Cl300 is resistant to fluoroquinolones due to the mutations found in the *gyrA* DNA gyrase and *parC* topoisomerase IV genes, leading to GyrA S81L and ParC S84L substitutions. These specific mutations are well-known to cause resistance to fluoroquinolones (e.g., nalidixic acid) in Gram-negative bacteria, including A. baumannii (22, 23).

pCl300 is cryptic and encodes a novel putative replication initiation protein that belongs to the Rep_3 family (PFAM01051). Its closest known Rep is RepAci3 encoded by p203 (GenBank accession number GU978997). An identical plasmid was found in A. baumannii strain ACN21 (GenBank accession number CP038648). ACN21 is recovered in India but also belongs to ST85_IP_, the same as Cl300.

### Cl300 is resistant to high levels of meropenem and amikacin.

MIC testing was used to examine whether the presence of *bla*_NDM_ and the multiplication of *aphA6* in Cl300 have led to higher levels of carbapenem and amikacin resistance. The meropenem MIC was found to be 512 mg/L, versus 16, 32. and 128 mg/L for D36 (with a single copy of *oxa23*), ACICU (carrying *oxa58*), and ABS201 (carrying *oxa24*), respectively. This signifies the contribution of a single copy of *bla*_NDM_ in Cl300 toward its extensive carbapenem resistance, given that the CLSI recommends the >8-mg/L (for both imipenem and meropenem) threshold to consider a strain as resistant.

The amikacin MIC for Cl300 was found to be >512 mg/L (versus 8 mg/L for A388 with a single *aphA6* copy), which is significant given the CLSI recommendation of ≥64-mg/L threshold for considering a strain as resistant. We also analyzed the Illumina read depth of the *aphA6* sequence relative to the *gyrA* gene, which is a housekeeping gene located in the chromosome. The sequence of the *gyrA* gene had a 30.28 coverage, while the aphA6 sequence read depth was 399.26 (SRA accession number SRR15725578). resulting in a 13.18-fold increase in sequence depth/coverage of *aphA6* relative to the chromosome, which correlates well with the presence of 14 *aphA6* copies observed in the final assembly (see below) and, hence, the high amikacin resistance level observed.

### Cl300 contains Tn*6924*, a novel Tn*7* family transposon with a *bla*_NDM_ and 14 copies of *aphA6*.

**FIG 1:**
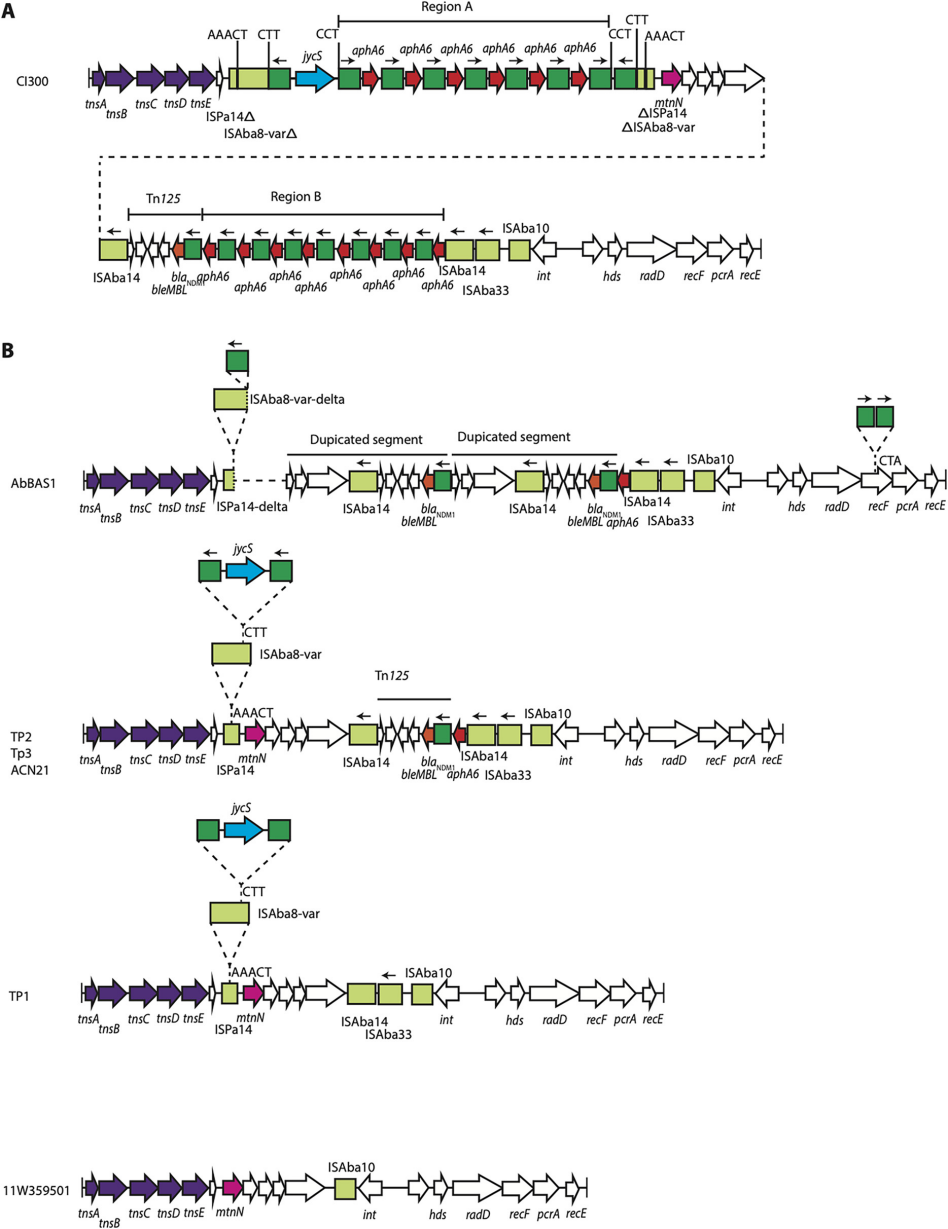
Tn*6924*. (A) Structure of Tn*6924* in Cl300 (GenBank accession no. CP082952); (B) structure in Acinetobacter baumannii strains AbBAS1 (GenBank accession no. CP065392), TP1 (GenBank accession no. CP056784), TP2 (GenBank accession no. CP060011), TP3 (GenBank accession no. CP060013), ACN21 (GenBank accession no. CP038644), and 11W359501 (GenBank accession no. CP041035). Copies of ISAba125 are shown as dark green boxes, and other insertion sequences are shown as light green boxes. Inverted repeats are shown as vertical bars, and the nucleotide sequences of target site duplications are shown above the line. The orientation and extent of genes are indicated by horizontal arrows. Transposition genes are colored purple, and antibiotic resistance genes are colored orange and red. Structures of known origin are labeled.

Analysis of the Cl300 genome showed that all 14 *aphA6* copies, as well as the *bla*_NDM_ gene, were clustered in a chromosomal region at the 3′ end of the *glmS* gene (locus id K9C16_00775 in GenBank accession number CP082952). We determined the boundaries of this element and found that it is bounded by 29-bp imperfect inverted repeats (IRs) and flanked by a 5-bp duplication of the target site (TGCCT at bases 92721 to 92725 in GenBank accession number CP082952). Examination of this region also indicated the presence of a set of genes encoding transposition proteins related to the TnsABCDE transposition proteins (29 to 50% amino acid identity; locus IDs K9C16_00750, K9C16_00755, K9C16_00760, K9C16_00765, and K9C16_00770 in GenBank accession number CP082952) encoded by the well-studied Tn*7* transposon (24). Recently, we described Tn*6171* in several A. baumannii strains, which is a Tn*7* family transposon that carries the fimsbactin siderophore systems (25). Analysis of the TnsABCDE proteins found in Cl300 showed that they share 86 to 94% amino acid identity with those encoded by Tn*6171*. In addition, several overlapping copies of the inner part of the IRs, equivalent to the binding sites for the transposase TnsB (26), were found within 200 bp of the transposon boundaries. All these properties are reminiscent of Tn*7* and its related transposons (often called Tn*7* family transposons), and the transposon in Cl300 was called Tn*6924*. Tn*6924* is a 76,190-bp novel Tn*7* family transposon (bases 92726 to 168915 in GenBank accession number CP082952) with a highly complex evolutionary history and a mosaic structure composed of multiple class I transposons and insertion sequences. The 14 copies of *aphA6* are in two separate locations in Tn*6924*, and each copy of *aphA6* is interspersed with copies of ISAba125 in a structure known as Tn*aphA6* (27). Six tandem duplications of Tn*aphA6* (region A in Fig. 1) are found inside an existing transposon comprised of ISAba125-*jycS*-ISAba125 and have generated a 3-bp target site duplication (CTT), suggesting this structure has arisen via traditional transposition followed by tandem duplication of the individual Tn*aphA6* unit. The second set of eight tandem duplications of Tn*aphA6* (region B in Fig. 1), adjacent to *bla*_NDM-1_ in their expected location in Tn*125*, appear to have duplicated in place, as there are no target site duplications detectable.

### Variants of Tn*6924* found in unrelated sequence types, including major global clones 1 and 2.

A search of the GenBank nonredundant database with the Tn*6924* sequence revealed that variants of Tn*6924* are present in seven other A. baumannii strains, including AbBAS1, 11W359501, TP1, TP2, TP3, ACN21, and AF401 (Table 1). There appear to be issues with the assembly of the AF401 sequence, and it will not be examined further. The remaining six strains belong to multiple sequence types (STs), including two (AbBAS1 and ACN21) belonging to ST85_IP_ (Table 1), as well as members of the major globally distributed clones GC1 (11W359501) (Table 1) and GC2 (TP1, TP2, and TP3) (Table 1). The variation in the structures (Fig. 1) is characterized by deletions, duplications, and acquisition of additional insertion sequences (IS). The structures in TP2, TP3, and ACN21 are identical to one another, and these likely represent an early evolutionary step of the structure seen in Cl300 prior to insertion of the *aphA6* tandem duplications in ISAba125-*jycS*-ISAba125 and prior to the second set of duplications arising. The structure in TP1 is identical to that in TP2/TP3/ACN21 except for a recombination between the two directly oriented copies of ISAba14, which has removed a 6,673-bp fragment containing *bla*_NDM-1_ and *aphA6.*

**TABLE 1:** General properties of strains containing Tn*6924* or its variants

Strain	Yr	Country	Source	ST_IP_	ST_OX_	GenBank acc. no.
Cl300	2015	Lebanon	TA^*a*^	85	1089	CP082952
ACN21	2018	India	Blood	85	1089	CP038644
AbBAS1	2019	Spain	Clinical	85	957	CP065392
11W359501	2015	UK	NK^*b*^	1	231	CP041035
TP1	2016	USA	Clinical	570^*c*^	1578	CP056784
TP2	2016	USA	Clinical	570	1578	CP060011
TP3	2016	USA	Clinical	570	1578	CP060013
AF401	2009	Mexico	Small colon	79	1974	CP018254

a TA, tracheal aspirate.

b NK, not known.

c ST570 is a single-locus variant (differs in *rpoB*) of ST2, represents global clone 2.

The structure in AbBAS1 is more complicated. An adjacent deletion originating from the first copy of ISAba125 in the ISAba125-*jycS-*ISAba125 transposon has extended into an open reading frame (ORF) downstream of *mtnN* and has deleted 17,032 bp relative to the structure in Cl300. There has also been a duplication of a 10,462-bp segment between the two copies of ISAba125, which has introduced a second copy of *bla*_NDM-1_. The structure in AbBAS1 has also acquired two additional copies of ISAba125 in a transposon-like structure, which has generated a 3-bp target site duplication.

The structure in 11W359501 is the simplest, and likely represents the original, or close to the original version of Tn*6924*. It does not contain any resistance genes and just a single IS, ISAba10.

### *sul2* and *msr*(E) are located in an IS*1008*-bounded pseudocompound transposon.

**FIG 2:**
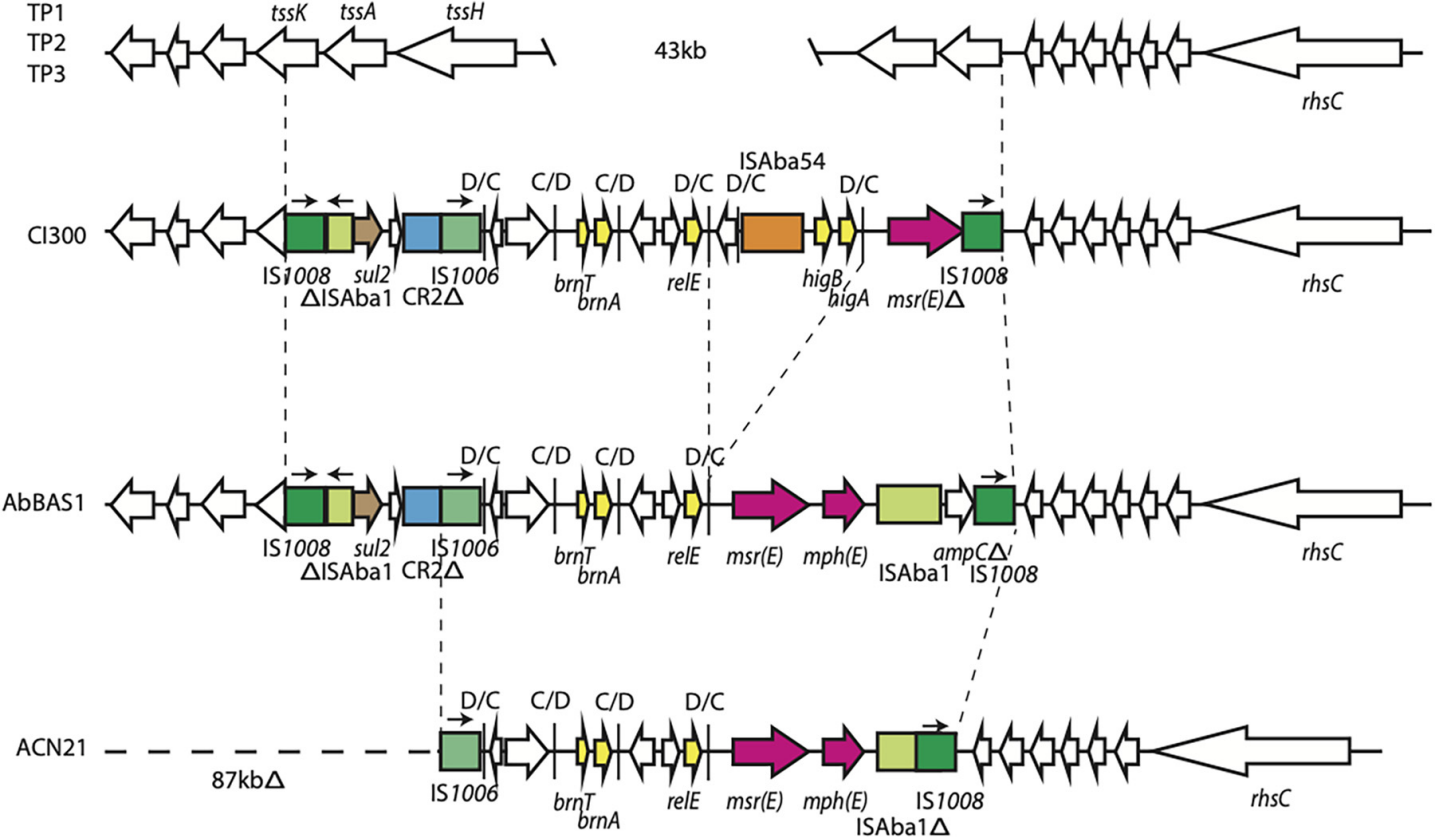
Structure of the IS*1008* pseudocompound transposon (PCT). Copies of IS*1008* are shown as dark green boxes, and other insertion sequences are shown as light green or orange boxes. Vertical black bars represent p*dif* sites, and the orientations of the p*dif* sites are shown above. The orientation and extent of genes are indicated by horizontal arrows. Known or predicted toxin-antitoxin genes are colored yellow, and the *msr*(E) and *mph*(E) antibiotic resistance genes are colored pink. Drawn to scale from GenBank accession numbers CP082952 (Cl300), CP056784 (TP1), CP060011 (TP2), CP060013 (TP3), CP065392 (AbBAS1), and CP038644 (ACN21).

The *sul2* and *msr*(E) genes were not accounted for in Tn*6924*. An analysis of the Cl300 genome revealed that they were in a 13.0-kb pseudocompound transposon (PCT) bounded by IS*1008* (Fig. 2), which has replaced 47.4 kb of the chromosome. This PCT includes multiple known p*dif* modules (28) containing toxin/antitoxin genes (yellow in Fig. 2) and the *msr*(E) resistance gene (pink in Fig. 2), the ISAba1-*sul2*-CR2 region associated with GI*sul2*, and additional IS. One of the IS, colored orange in Fig. 2, is novel and has been submitted to the ISFinder database under ISAba54. ISAba54 is related to ISAlw22 in the insertion sequence not classified yet family (ISNCY), sharing 89% nucleotide identity, and has generated a 5-bp target site duplication (TSD). The *msr*(E)-*mph*(E) *dif* module has been truncated by IS*1008*, removing *mph*(E) and truncating the 3′ end of *msr*(E). The remaining *dif* modules, containing the *brnTA*, *relE*, and *higAB* toxin/antitoxin genes, are either identical or related (∼5% nucleotide difference) to known *dif* modules (28).

The GenBank nonredundant database was searched with the left-hand and right-hand boundaries of this PCT. The only other genomes containing this PCT at this chromosomal location are AbBAS1 and ACN21, which also contained Tn*6924*. The PCT is not found in the other Tn*6924-*containing strains (TP1/TP2/TP3 or 11W359501). The PCT in AbBAS1 (Fig. 2) is 14.4 kb and is very similar to the structure in Cl300. A recombination between two D and C sites has removed two *dif* modules, including the *higAB* module. The *msr*(E)-*mph*(E) module is more complete than in Cl300, with both genes intact, but an ISAba1 has removed the last 38 bp of the module. The ISAba1 is associated with a truncated copy of the *ampC* gene, with IS*1008* removing 273 bp from the 3′ end of *ampC*. It is not clear how this structure has arisen, but it is likely that it may be the progenitor of the structure seen in Cl300 given that more of the *msr*(E)-*mph*(E) module is intact. The PCT in ACN21 (Fig. 2) is 11.5 kb and differs from the structure seen in AbBAS1 by two IS-mediated deletions. The IS*1008* at the right-hand end has been deleted into the ISAba1, removing the remainder of *ampC*. A deletion arising from the internal copy of IS*1006* has removed the entire left-hand side of the island, including the fragment originating from GI*sul2*, and has deleted 87 kb of the adjacent backbone.

### Phylogenetic analysis of ST85_IP_ genomes reveals multiple lineages.

**FIG 3:**
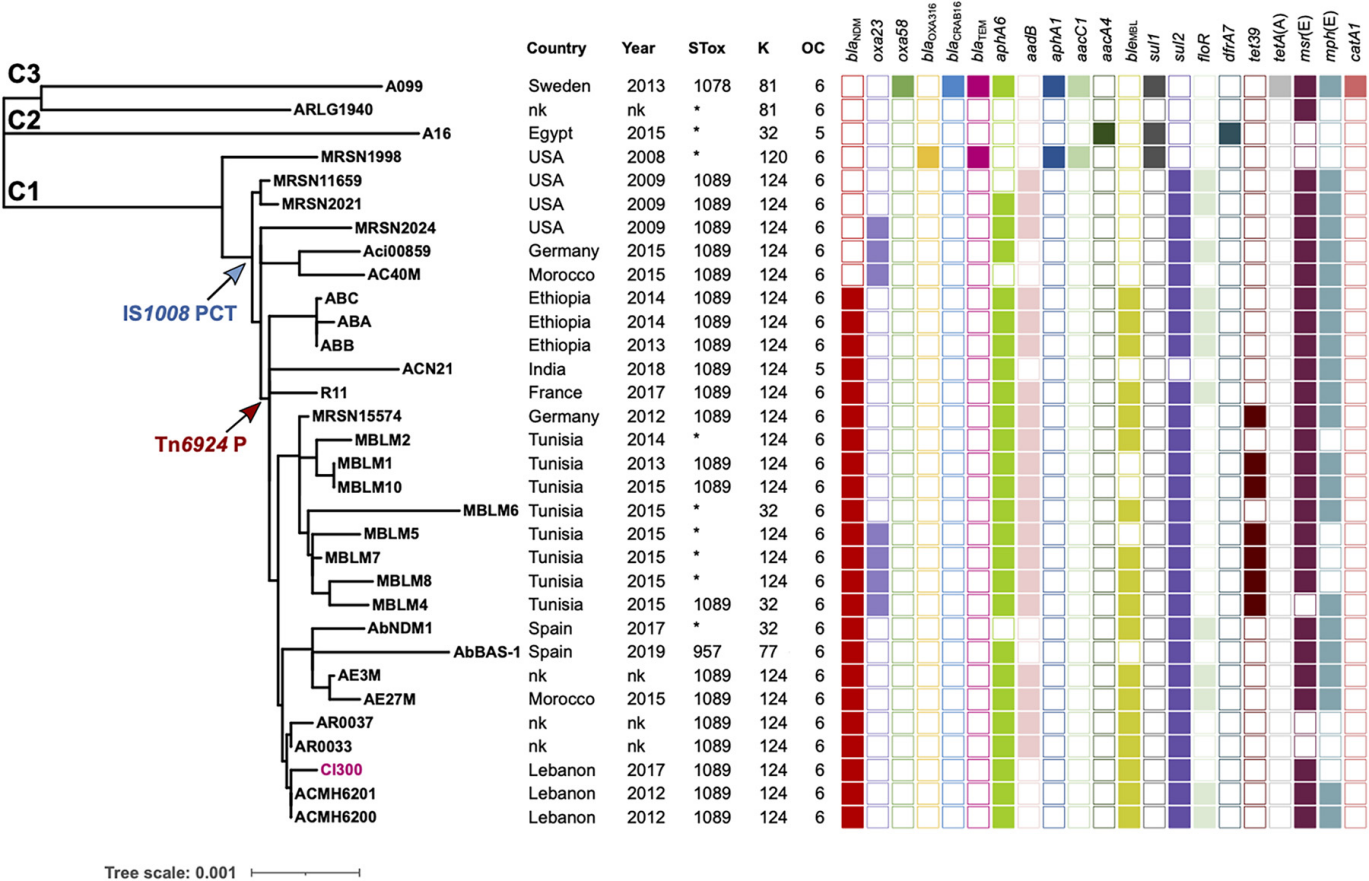
Phylogenetic tree of ST85_IP_ genomes constructed using the whole-genome alignment of ST85_IP_ strains. C1-3 indicate clades 1, 2, and 3. Blue and red arrows marked IS*1008* PCT and Tn*6926*P indicate the entry of the pseudocompound transposon (PCT) and the precursor of Tn*6926* in ST85_IP_, respectively. Color-coded filled boxes (on the right) indicate the presence/absence of antibiotic resistance genes in ST85_IP_ genomes, and the middle panel includes genomes’ metadata. The K and OC columns indicate the capsular and outer core surface polysaccharides. STs indicated with an asterisk are novel and could not be determined. Nk indicates not known.

To examine the phylogenetic relationship of Cl300 to other ST85_IP_ strains, all publicly available ST85_IP_ genomes (31 genomes as of July 2021, including Cl300) were downloaded from GenBank and used to construct a whole-genome sequence phylogenetic tree (Fig. 3). All 31 genomes were screened for their important features such as antibiotic resistance genes, as well as K and OC surface polysaccharide loci (Table 2). ST85_IP_ genomes are from a diverse geographical region including several Middle Eastern (Lebanon), African (Egypt and Tunisia), and European countries. A single strain (ACN21) was recovered in India. The four Multidrug-Resistant Organism Repository and Surveillance Network (MRSN) strains recovered in the United States are military strains and are likely to have been imported from the Middle East region. All but one, ARLG1940, contain several antibiotic resistance genes (Table 2), such as those conferring resistance to carbapenems, aminoglycosides, tetracycline, and macrolides, indicating resistance to a wide range of clinically important antibiotics (Table 2). Most ST85_IP_ strains belong to ST1089_OX_. However, one belongs to ST1087_OX_ and one to ST957_OX_, and eight strains belong to novel sequence types. Analysis of the K and OC surface polysaccharides indicated that all but two ST85_IP_ genomes encode the OCL6 outer core locus. ACN21 and A15 contain OCL5. Although the majority of strains contained the KL124 capsular locus, a few strains encode KL32, KL81, or KL120.

**TABLE 2:** Properties of ST85_IP_ strains

Strain	Yr	Country	Source	ST_OX_	KL	OC	Antibiotic resistance genes	GenBank acc. no.^*d*^
Cl300	2015	Lebanon	TA^*a*^	1089	124	6	*aphA6* (14x), *bla*_NDM_, *ble*_MBL_, *sul2*, *msr*(E)	CP082952
A16	2015	Egypt	NK^*b*^	^*c*^	32	5	*sul1*, *dfrA7*, *aacA4*	JACSTQ
ACMH-6200	2012	Lebanon	Wound	1089	124	6	*msr-mph*(E), *floR*, *sul2*, *bleMBL*, *bla*_NDM_, *aphA6*	LKMA
ACMH-6201	2012	Lebanon	Wound	1089	124	6	*msr-mph*(E), *floR*, *ble*_MBL_, *bla*_NDM_, *sul2*, *aphA6*	LKMB
A099	2013	Sweden	LLL^*e*^	1078	81	6	*catA1* (2x), *tetA* (3x), *sul1* (2x), *msr-mph*(E) (2x), *oxa58* (2x), *aacC1* (3x), *bla*_TEM_ (2x), *aphA1*, *bla*_CARB16_ (2x), *aphA6*	DADARN
AR_0037	NK	NK	NK	1089	124	6	*aphA6*, *bla*_NDM_, *ble*_MBL_, *sul2*, *aadB* (2x)	MPBX
AR_0033	NK	NK	NK	1089	124	6	*aphA6*, *bla*_NDM_, *ble*_MBL_, *sul2*, *aadB* (2x)	MPCA
AB-A	2014	Ethiopia	Wound	1089	124	6	*ble*_MBL_, *bla*_NDM_, *sul2*, *floR*, *msr-mph*(E), *aadB*, *aphA6*	LWSM
AB-C	2014	Ethiopia	Wound	1089	124	6	*floR*, *sul2*, *aadB*, *msr-mph*(E), *bla*_NDM_, *ble*_MBL_, *aphA6*	LWSO
AB-B	2013	Ethiopia	Wound	1089	124	6	*aadB*, *msr-mph*(E), *floR*, *ble*_MBL_, *bla*_NDM_, *sul2*, *aphA6*	LWSN
ARLG1940	NK	NK	NK	1078	81	6	*msr*(E)	NGIM
AE27M	2015	Morocco	Hospital ventilator	1089	124	6	*msr-mph*(E), *aadB*, *bla*_NDM_, *ble*_MBL_, *floR*, *sul2*, *aphA6*	FWWO
AC40M	2015	Morocco	Anal margin	1089	124	6	*msr-mph*(E), *sul2*, *oxa23*	FWYN
AE3M	NK	NK	NK	1089	124	6	*msr-mph*(E), *sul2*, *aadB*, *ble*_MBL_, *bla*_NDM_, *aphA6*, *floR*	FWFB
MBL_M1	2013	Tunisia	Urine	1089	124	6	*aadB*, *tet39*, *msr-mph*(E), *aphA6*, *sul2*, *bla*_NDM1_	MWTR
MBL_M4	2015	Tunisia	Blood	1089	32	6	*aadB*, *tet39*, *oxa23*, *bla*_NDM_, *ble*_MBL_, *mph*(E), *sul2*, *aphA6*	MWTU
MBL_M5	2015	Tunisia	Urine	^*c*^	124	6	*aadB*, *tet39*, *aphA6* (2x), *bla*_NDM1_, *msr*(E), *oxa23*, *sul2*	MWTV
MBL_M6	2015	Tunisia	Urine	^*c*^	32	6	*ble*_MBL_, *bla*_NDM1_, *aadB*, *aphA6*, *sul2*, *msr-mph*(E)	MWTW
MBL_M2	2014	Tunisia	Urine	^*c*^	124	6	*aadB*, *bla*_NDM1_, *ble*_MBL_, *aphA6*, *sul2*, *msr*(E)	MWTS
MBL_M8	2015	Tunisia	Urine	^*c*^	124	6	*tet39*, *aadB*, *aphA6*, *aphA6*, *oxa23*, *ble*_MBL_, *bla*_NDM1_, *msr*(E), *sul2*	MWTY
MBL_M7	2015	Tunisia	Blood	^*c*^	124	6	*aadB*, *tet39*, *oxa23*, *ble*_MBL_, *bla*_NDM,_ *sul2*, *aphA6*(2x), *msr*(E)	MWTX
MBL_M10	2015	Tunisia	Blood	1089	124	6	*aadB*, *tet39*, *msr-mph*(E), *aphA6*, *sul2*, *bla*_NDM_	MWUA
R11	2017	Tahiti	NK	1089	124	6	*aadB*, *floR*, *bla*_NDM_, *ble*_MBL_, *sul2*, *msr*(E), *mph*(E), *aphA6*	QKWE
MRSN11659	2009	USA	NK	1089	124	6	*msr-mph*(E), *aadB*, *sul2*, *floR*	AAYNPT
MRSN1998	2008	USA	Wound	^*c*^	120	6	*bla*_OXA316_, *sul1*, *aacC1*, *bla*_TEM_, *aphA1*	AAYNTJ
MRSN2021	2009	USA	NK	1089	124	6	*sul2*, *msr-mph*(E), *aadB*, *floR*, *aphA6*	AAYNRB
MRSN2024	2009	USA	Wound	1089	124	6	*oxa23*, *sul2*, *msr-mph*(E), *aadB*, *aphA6*	AAYNNH
Ab-NDM-1	2017	Spain	Rectal swab	^*c*^	32	6	*sul2*, *ble*_MBL_, *bla*_NDM_, *floR*, *msr-mph*(E)	QBBY
ACN21	2018	India	Blood	1089	124	5	*msr-mph*(E), *bla*_NDM_, *aphA6*	CP038644
Aci00859	2015	Germany	Wound	1089	124	6	*msr-mph*(E), *sul2*, *floR*, *oxa23*, *aphA6*	VAGE
MRSN15574	2012	Germany	Respiratory	1089	124	6	*msr-mph*(E), *sul2*, *tet39*, *aadB*, *bla*_NDM_, *ble*_MBL_, *aphA6*	VHGP

a TA, tracheal aspirate.

b NK, not known.

c Could not be determined (novel, single-locus variants of either 1089 or 1078).

d Draft genomes’ accession numbers include a set of letters followed by 00000000. Here, short forms (letters only) have been used.

e Lung’s Lower Lobe sample.

The whole-genome phylogenetic tree showed that the ST85_IP_ genomes belong to three different clades with the major clade (C1) containing 28 strains, the second clade (C2) only has 1 strain, and the last clade (C3) has 2 strains (Fig. 3). Each clade consisted of multiple sublineages. Strains from the same geographical regions appear to cluster together, suggesting an *in situ* evolution of lineages in each niche. Notably, 22 isolates contain the *bla*_NDM_ metallo-β-lactamase gene, with all but one also containing the *aphA6* amikacin resistance gene. Analysis of these draft genomes predicted that both *bla*_NDM_ and *aphA6* are in Tn*6924* or its variants, indicating the significance of the transposon in bringing in and the spread of these important resistance genes. Analysis of the phylogenetic tree combined with the distribution of antibiotic resistance genes revealed the insertion point of both Tn*6924* and the IS*1008* flanked *sul2-msr*(E)*-mph*(E) PCT (blue and gray arrows in Fig. 3). The acquisition of the IS*1008* flanked *sul2-msr*(E)*-mph*(E) PCT appears to be an earlier event relative to the acquisition of Tn*6924*.

## DISCUSSION

In A. baumannii, carbapenem resistance is mainly due to the production of oxacillin carbapenemases (e.g., OXA-23, OXA-24, and OXA-58), while other acquired carbapenem resistance genes are uncommon (2). The emergence of CRAb strains that carry the *bla*_NDM_ gene, a gene frequently found in Escherichia coli and Klebsiella pneumoniae, is therefore a major public health concern given that *bla*_NDM_ confers resistance not only to carbapenems but also to all β-lactams (excluding aztreonam) (29). While the majority of the global spread of CRAb is due to two main global clones, GC1 and GC2, several emerging sequence types (STs) have also been reported (2), such as those belonging to ST10, ST25, and ST85_IP_ (2, 16, 17, 20). ST85 strains, now reported in Lebanon, Tunisia, Egypt, Spain, Turkey, and Germany, have been known to carry the *bla*_NDM_ carbapenem resistance gene (11–17, 19–21). Here, we completed and analyzed the genome of Cl300, a carbapenem-resistant ST85_IP_ strain recovered in Lebanon, and determined the context of its resistance genes, including *bla*_NDM_ and 14 copies of the amikacin resistance gene *aphA6.* We showed that in Cl300, the *bla*_NDM_ and 14 *aphA6* genes are present in a novel Tn*7* family transposon called Tn*6924*. Tn*6924* has a compound structure and a complex *in situ* evolutionary history that includes many IS/transposon insertion/deletion and multiplication events. It is likely that high amikacin selective pressure has facilitated the amplification of Tn*aphA6*, leading to the current structure and, therefore, high levels of amikacin resistance (MIC > 512 mg/L). Analyzing the genomes of all publicly available ST85_IP_ genomes predicted the presence of Tn*6924* and/or its variants in a subclade of the main phylogenetic clade (22 strains) (blue arrow marked “Tn” in Fig. 3). In addition, several variants of Tn*6924* were found in a small set of diverse strains, some of which belong to the major global clones GC1 and GC2 (Table 1), the most encountered and, indeed, the most successful clones of A. baumannii. The presence of Tn*6924/*variants in these strains will alarmingly increase the possibility of further spreading *bla*_NDM_ and *aphA6*.

Moreover, we determined the structure of an IS*1008* PCT carrying the sulfonamide *sul2* and macrolide *msr*(E) resistance genes in Cl300 and in the other two complete ST85_IP_ genomes (ACN21 and AbBAS-1) (Fig. 2). Analysis performed here predicted a complex *in situ* evolution of the IS*1008* PCT with its central fragment originating from small A. baumannii plasmids previously described (28). Interestingly, all but one strain (MRSN1998) in the main ST85_IP_ clade are also predicted to include a variant of the IS*1008* PCT. Phylogenetic analysis, combined with the presence of resistance genes, indicates an earlier acquisition of IS*1008* PCT than the entry of the Tn*6924* precursor, which is in line with the history of antibiotic usage where the introduction of older antibiotics precedes the last-line carbapenem antibiotics.

In summary, Tn*7* and its variants represent a diverse family of transposons that carry a wide variety of genes, including antibiotic resistance genes that contribute to the success of diverse organisms (30). Tn*6924* represents a Tn*7* family transposon that contributes to the success of a diverse set of ST85_IP_ strains. Although it appears that ST85s at this stage are still limited to the Middle East region, North Africa, and a few European countries (with only a single genome from India and all MRSN U.S. strains likely imported from the Middle East), outbreaks and sporadic cases caused by these strains should be fully characterized and tracked to identify sources and potential transmission routes.

## MATERIALS AND METHODS

### Bacterial strain and publicly available genome sequence data used in this study.

A. baumannii Cl300 was recovered in 2015 from a tracheal aspirate of a male patient in Tripoli Governmental Hospital, Tripoli, North Lebanon. Cl300 was previously reported as part of a study investigating the epidemiology of A. baumannii strains isolated from different hospitals in Lebanon (11).

To examine the relationship of Cl300 to other ST85_IP_ genomes, >4,000 A. baumannii genomes publicly available in GenBank (https://www.ncbi.nlm.nih.gov/genome/403) were screened for ST85_IP_ strains using the mlst program publicly available at https://github.com/tseemann/mlst. Thirty-one ST85_IP_ genomes (2 complete and 29 draft genomes) were found and included in phylogenetic analysis of this study.

### Antibiotic susceptibility and resistance testing.

The antibiotic resistance profile was determined using the calibrated dichotomous sensitivity (CDS) disk diffusion method as described previously (31). Resistance profiles were interpreted according to the Clinical and Laboratory Standards Institute (CLSI) guidelines for Acinetobacter spp. (32) and CDS disk diffusion assay (http://cdstest.net) (31) when a CLSI breakpoint for Acinetobacter spp. was not available (e.g., for netilmicin, streptomycin, spectinomycin, sulfamethoxazole, nalidixic acid, and rifampicin). MICs of amikacin, meropenem, and colistin were determined using the standard broth microdilution method as described elsewhere (33). A. baumannii A388 (carrying a single copy of *aphA6*) was used as a control in amikacin MIC assay. A. baumannii strains D36 (carrying *oxa23*) and ACICU (containing *oxa58*) were used as controls in meropenem MIC assays.

### Whole-genome sequencing, assembly, and annotation.

Cl300 cells were grown overnight at 37°C in LB inoculated from a single colony. Genomic DNA was isolated using the DNeasy UltraClean microbial kit (Qiagen, Germantown, MD, USA), and sequencing was performed using Illumina MiSeq and Oxford Nanopore (MinION). Library preparation and barcoding for Illumina MiSeq and MinION (Oxford Nanopore Technologies, Oxford, UK) sequencing was performed as described previously (34). Illumina sequencing generated 5,865,134 paired-end short reads with 150-fold coverage and an average length of 250 bp, and MinION generated a total of 37,640 reads with an *N*_50_ of 15.5 kbp and ∼50-fold coverage.

The FastQC (v.0.11.9) program (https://www.bioinformatics.babraham.ac.uk/projects/fastqc/) was used (default setting) to check the quality of Illumina reads (e.g., per-base sequence quality of >38, per-base sequence content of <10%, etc.). Filtlong (v.0.2.0) (https://github.com/rrwick/Filtlong) was used to filter the MinION reads with low quality by discarding any read shorter than 1 kbp (--min_length 1000). In addition, PHRED read quality scores assigned in the FASTQ file during basecalling were used by Filtlong to discard the worst 10% of reads measured by bp rather than read counts. The latter was done using the parameter —keep_percent 90. The high-quality Illumina and MinION reads were assembled *de novo* using a hybrid assembly approach with the Unicycler program (v0.4.7) using default settings (35).

Protein coding, rRNA, and tRNA gene sequences were annotated using Prokka (36), followed by manual annotations of resistance genes, insertion sequences, and surface polysaccharide loci using ResFinder (https://cge.cbs.dtu.dk/services/ResFinder/), ISFinder (https://www-is.biotoul.fr/), BLASTp (https://blast.ncbi.nlm.nih.gov/Blast.cgi?PAGE=Proteins), Pfam (http://pfam.xfam.org/), and Kaptive (https://kaptive-web.erc.monash.edu/) (37) searches. Multilocus sequence types (MLSTs) in the Institut Pasteur and Oxford schemes (http://pubmlst.org/abaumannii/) were determined from the genome sequence data. Figures were drawn to scale using SnapGene (v.5.2.4) and InkScape (v.1.0).

To examine the relationship of Cl300 to other ST85_IP_ genomes, all publicly available ST85_IP_ genomes (31 genomes as of July 2021) were downloaded from GenBank and used to construct a recombination-free whole-genome phylogenetic tree as described previously (38). The phylogenetic tree was visualized and annotated using the iTOL online phylogenetic tree visualization tool (https://itol.embl.de/).

### Data availability.

The complete genome and plasmid sequences of Cl300 have been deposited in the GenBank/EMBL/DDBJ database and are publicly available under the accession numbers CP082952 (chromosome) and CP082953 (pCl300). Illumina short reads and MinION long reads are also deposited under the Sequence Read Archive (SRA) accession numbers SRR15725578 and SRR15725577, respectively.

## References

[1] Harding CM, Hennon SW, Feldman MF. 2018. Uncovering the mechanisms of *Acinetobacter baumannii* virulence. Nat Rev Microbiol 16:91–102. doi:10.1038/nrmicro.2017.148. 29249812 PMC6571207

[2] Hamidian M, Nigro SJ. 2019. Emergence, molecular mechanisms and global spread of carbapenem-resistant *Acinetobacter baumannii*. Microb Genom 5:e000306. doi:10.1099/mgen.0.000306. PMC6861865 31599224

[3] Higgins PG, Hagen RM, Kreikemeyer B, Warnke P, Podbielski A, Frickmann H, Loderstädt U. 2021. Molecular epidemiology of carbapenem-resistant *Acinetobacter baumannii* isolates from Northern Africa and the Middle East. Antibiotics 10:291. doi:10.3390/antibiotics10030291. 33799540 PMC8002098

[4] Zarrilli R, Pournaras S, Giannouli M, Tsakris A. 2013. Global evolution of multidrug-resistant *Acinetobacter baumannii* clonal lineages. Int J Antimicrob Agents 41:11–19. doi:10.1016/j.ijantimicag.2012.09.008. 23127486

[5] da Silva KE, Maciel WG, Croda J, Cayô R, Ramos AC, de Sales RO, Kurihara MNL, Vasconcelos NG, Gales AC, Simionatto S. 2018. A high mortality rate associated with multidrug-resistant *Acinetobacter baumannii* ST79 and ST25 carrying OXA-23 in a Brazilian intensive care unit. PLoS One 13:e0209367. doi:10.1371/journal.pone.0209367. 30592758 PMC6310363

[6] Hamidian M, Hall RM. 2016. The resistance gene complement of D4, a multiply antibiotic-resistant ST25 *Acinetobacter baumannii* isolate, resides in two genomic islands and a plasmid. J Antimicrob Chemother 71:1730–1732. doi:10.1093/jac/dkw041. 26944923

[7] Hamidian M, Holt KE, Hall RM. 2015. Genomic resistance island AGI1 carrying a complex class 1 integron in a multiply antibiotic-resistant ST25 *Acinetobacter baumannii* isolate. J Antimicrob Chemother 70:2519–2523. doi:10.1093/jac/dkv137. 26023211

[8] Montaña S, Vilacoba E, Fernandez JS, Traglia GM, Sucari A, Pennini M, Iriarte A, Centron D, Melano RG, Ramírez MS. 2020. Genomic analysis of two *Acinetobacter baumannii* strains belonging to two different sequence types (ST172 and ST25). J Glob Antimicrob Resist 23:154–161. doi:10.1016/j.jgar.2020.09.006. 32966912

[9] Rodríguez CH, Nastro M, Famiglietti A. 2018. Carbapenemases in *Acinetobacter baumannii*. Review of their dissemination in Latin America. Rev Argent Microbiol 50:327–333. doi:10.1016/j.ram.2017.10.006. 29548732

[10] Sahl JW, Del Franco M, Pournaras S, Colman RE, Karah N, Dijkshoorn L, Zarrilli R. 2015. Phylogenetic and genomic diversity in isolates from the globally distributed *Acinetobacter baumannii* ST25 lineage. Sci Rep 5:15188. doi:10.1038/srep15188. 26462752 PMC4604477

[11] Al Atrouni A, Hamze M, Jisr T, Lemarié C, Eveillard M, Joly-Guillou ML, Kempf M. 2016. Wide spread of OXA-23-producing carbapenem-resistant *Acinetobacter baumannii* belonging to clonal complex II in different hospitals in Lebanon. Int J Infect Dis 52:29–36. doi:10.1016/j.ijid.2016.09.017. 27663910

[12] Bakour S, Olaitan AO, Ammari H, Touati A, Saoudi S, Saoudi K, Rolain JM. 2015. Emergence of colistin- and carbapenem-resistant *Acinetobacter baumannii* ST2 clinical isolate in Algeria: first Case report. Microb Drug Resist 21:279–285. doi:10.1089/mdr.2014.0214. 25588125

[13] Bonnin RA, Cuzon G, Poirel L, Nordmann P. 2013. Multidrug-resistant *Acinetobacter baumannii* clone, France. Emerg Infect Dis 19:822–823. doi:10.3201/eid1905.121618. 23697750 PMC3647512

[14] Cheikh HB, Domingues S, Silveira E, Kadri Y, Rosário N, Mastouri M, Da Silva GJ. 2018. Molecular characterization of carbapenemases of clinical *Acinetobacter baumannii*-*calcoaceticus* complex isolates from a university hospital in Tunisia. Biotech 8:297. doi:10.1007/s13205-018-1310-3. PMC6021275 29963357

[15] Decousser JW, Jansen C, Nordmann P, Emirian A, Bonnin RA, Anais L, Merle JC, Poirel L. 2013. Outbreak of NDM-1-producing *Acinetobacter baumannii* in France, January to May 2013. Euro Surveill 18:20547. doi:10.2807/1560-7917.es2013.18.31.20547. 23929226

[16] Fernández-Cuenca F, Pérez-Palacios P, Galán-Sánchez F, López-Cerero L, López-Hernández I, López Rojas R, Arca-Suárez J, Díaz-de Alba P, Rodríguez Iglesias M, Pascual A. 2020. First identification of *bla*_NDM-1_ carbapenemase in *bla*_OXA-94_-producing *Acinetobacter baumannii* ST85 in Spain. Enferm Infecc Microbiol Clin (Engl Ed) 38:11–15. doi:10.1016/j.eimc.2019.03.008. 31060865

[17] Heydari F, Mammina C, Koksal F. 2015. NDM-1-producing *Acinetobacter baumannii* ST85 now in Turkey, including one isolate from a Syrian refugee. J Med Microbiol 64:1027–1029. doi:10.1099/jmm.0.000132. 26296677

[18] Jeannot K, Diancourt L, Vaux S, Thouverez M, Ribeiro A, Coignard B, Courvalin P, Brisse S. 2014. Molecular epidemiology of carbapenem non-susceptible *Acinetobacter baumannii* in France. PLoS One 9:e115452. doi:10.1371/journal.pone.0115452. 25517732 PMC4269441

[19] Rafei R, Pailhoriès H, Hamze M, Eveillard M, Mallat H, Dabboussi F, Joly-Guillou ML, Kempf M. 2015. Molecular epidemiology of *Acinetobacter baumannii* in different hospitals in Tripoli, Lebanon using *bla*_OXA-51-like_ sequence based typing. BMC Microbiol 15:103. doi:10.1186/s12866-015-0441-5. 25976451 PMC4432822

[20] Jaidane N, Naas T, Oueslati S, Bernabeu S, Boujaafar N, Bouallegue O, Bonnin RA. 2018. Whole-genome sequencing of NDM-1-producing ST85 *Acinetobacter baumannii* isolates from Tunisia. Int J Antimicrob Agents 52:916–921. doi:10.1016/j.ijantimicag.2018.05.017. 29857033

[21] Mosqueda N, Espinal P, Cosgaya C, Viota S, Plasensia V, Alvarez-Lerma F, Montero M, Gómez J, Horcajada JP, Vila J, Roca I. 2013. Globally expanding carbapenemase finally appears in Spain: nosocomial outbreak of *Acinetobacter baumannii* producing plasmid-encoded OXA-23 in Barcelona, Spain. Antimicrob Agents Chemother 57:5155–5157. doi:10.1128/AAC.01486-13. 23877694 PMC3811394

[22] Vila J, Ruiz J, Goñi P, Marcos A, Jimenez de Anta T. 1995. Mutation in the *gyrA* gene of quinolone-resistant clinical isolates of *Acinetobacter baumannii*. Antimicrob Agents Chemother 39:1201–1203. doi:10.1128/AAC.39.5.1201. 7625818 PMC162713

[23] Vila J, Ruiz J, Goñi P, Jimenez de Anta T. 1997. Quinolone-resistance mutations in the topoisomerase IV *parC* gene of *Acinetobacter baumannii*. J Antimicrob Chemother 39:757–762. doi:10.1093/jac/39.6.757. 9222045

[24] Craig NL. 1996. Transposon Tn*7*. Curr Top Microbiol Immunol 204:27–48. doi:10.1007/978-3-642-79795-8_2. 8556868

[25] Hamidian M, Hall RM. 2021. Dissemination of novel Tn*7* family transposons carrying genes for synthesis and uptake of fimsbactin siderophores among *Acinetobacter baumannii* isolates. Microb Genom 7:mgen000548. doi:10.1099/mgen.0.000548. PMC8190619 33749577

[26] Arciszewska LK, Craig NL. 1991. Interaction of the Tn*7*-encoded transposition protein TnsB with the ends of the transposon. Nucleic Acids Res 19:5021–5029. doi:10.1093/nar/19.18.5021. 1656385 PMC328805

[27] Nigro SJ, Post V, Hall RM. 2011. Aminoglycoside resistance in multiply antibiotic-resistant *Acinetobacter baumannii* belonging to global clone 2 from Australian hospitals. J Antimicrob Chemother 66:1504–1509. doi:10.1093/jac/dkr163. 21586593

[28] Blackwell GA, Hall RM. 2017. The *tet39* determinant and the *msrE*-*mphE* genes in *Acinetobacter* plasmids are each part of discrete modules flanked by inversely oriented p*dif* (XerC-XerD) sites. Antimicrob Agents Chemother 61:e00780-17. doi:10.1128/AAC.00780-17. 28533235 PMC5527579

[29] Poirel L, Bonnin RA, Boulanger A, Schrenzel J, Kaase M, Nordmann P. 2012. Tn*125*-related acquisition of *bla*_NDM-like_ genes in *Acinetobacter baumannii*. Antimicrob Agents Chemother 56:1087–1089. doi:10.1128/AAC.05620-11. 22143526 PMC3264265

[30] Parks AR, Peters JE. 2009. Tn*7* elements: engendering diversity from chromosomes to episomes. Plasmid 61:1–14. doi:10.1016/j.plasmid.2008.09.008. 18951916 PMC2614081

[31] Bell SM, Smith DD. 1975. The CDS disc method of antibiotic sensitivity testing (calibrated dichotomous sensitivity test). Pathology 7:1–48. doi:10.3109/00313027509082602. 772573

[32] Clinical and Laboratory Standards Institute. 2019. Performance standards for antimicrobial susceptibility testing; 29th informational supplement. Clinical and Laboratory Standards Institute, Wayne, PA.

[33] Wiegand I, Hilpert K, Hancock RE. 2008. Agar and broth dilution methods to determine the minimal inhibitory concentration (MIC) of antimicrobial substances. Nat Protoc 3:163–175. doi:10.1038/nprot.2007.521. 18274517

[34] Wick RR, Judd LM, Gorrie CL, Holt KE. 2017. Completing bacterial genome assemblies with multiplex MinION sequencing. Microb Genom 3:e000132. doi:10.1099/mgen.0.000132. 29177090 PMC5695209

[35] Wick RR, Judd LM, Gorrie CL, Holt KE. 2017. Unicycler: resolving bacterial genome assemblies from short and long sequencing reads. PLoS Comput Biol 13:e1005595. doi:10.1371/journal.pcbi.1005595. 28594827 PMC5481147

[36] Seemann T. 2014. Prokka: rapid prokaryotic genome annotation. Bioinformatics 30:2068–2069. doi:10.1093/bioinformatics/btu153. 24642063

[37] Wyres KL, Cahill SM, Holt KE, Hall RM, Kenyon JJ. 2020. Identification of *Acinetobacter baumannii* loci for capsular polysaccharide (KL) and lipooligosaccharide outer core (OCL) synthesis in genome assemblies using curated reference databases compatible with Kaptive Microb Genom 6:e000339. doi:10.1093/bioinformatics/btu153. PMC7200062 32118530

[38] Holt K, Kenyon JJ, Hamidian M, Schultz MB, Pickard DJ, Dougan G, Hall R. 2016. Five decades of genome evolution in the globally distributed, extensively antibiotic-resistant *Acinetobacter baumannii* global clone 1. Microb Genom 2:e000052. doi:10.1099/mgen.0.000052. 28348844 PMC5320584

