# Supplementary material for: Variants of Tn6924, a Novel Tn7 Family Transposon Carrying the blaNDM Metallo-β-Lactamase and 14 Copies of the aphA6 Amikacin Resistance Genes Found in Acinetobacter baumannii
Source: Microbiol Spectr. 2022 Jan 12;10(1):e01745-21. doi: 10.1128/spectrum.01745-21 (PMC8754128; doi:10.1128/spectrum.01745-21)
Supplement: SUPPLEMENTAL FILE 1 — Supplemental material. Download SPECTRUM01745-21_Supp_1_seq9.pdf, PDF file, 0.01 MB [file spectrum01745-21_supp_1_seq9.pdf]

**Table S1.** Antibiotic susceptibility profiles of Cl300 and A388 (control).

| Isolate | IPM[a] | MEM | AMP | CTX | SAM | CAZ | S | AK | NET | CN | SH | TOB | N | K | RL | CRO | NA | CIP | W | RD | FFC | TE | C |
|---|---|---|---|---|---|---|---|---|---|---|---|---|---|---|---|---|---|---|---|---|---|---|---|
| Cl300 | 8 (R) [b] | 0(R) | 0(R) | 0(R) | 20 (I) | 0(R) | 11(R) | 8(R) | 14(S) | 10(R) | 7(R) | 15(S) | 9(R) | 0(R) | 9(R) | 0(R) | 0(R) | 0(R) | 0(R) | 20(S) | 0(R) | 18(S) | 0(R) |
| A388 | 17(S) | 8(R) | 0(R) | 0(R) | 15 (S) | 0(R) | 11(R) | 9(R) | 0(R) | 11(R) | 8(R) | 9(R) | 8(R) | 0(R) | 9(R) | 0(R) | 15(S) | 22(S) | 9(R) | 26(S) | 12(I) | 8(R) | 12(I) |

[a] Abbreviations: IPM: Imipenem, MEM: Meropenem, AMP: Ampicillin, CTX: Cefotaxime, SAM: Ampicillin-Sulbactam, CAZ: Ceftazidime, S: Streptomycin, AK: Amikacin, NET: Netilmicin, CN: Gentamicin, SH: Spectpnomycin, TOB: Tobramycin, N: Neomycin, K: Kanamycin, RL: Sulphamethoxazole, CRO: Ceftriaxone, NA: Nalidixic acid, CIP: Ciprofloxacin, W: Trimethoprim, RD: Rifampin, FFC: Florfenicol, TE: Tetracycline, C: Chloramphenicol.

[b] numbers indicate diameters of inhibition growth ring in millimetres**.**
